# The Limitations of Large Language Models for Understanding Human Language and Cognition

**DOI:** 10.1162/opmi_a_00160

**Published:** 2024-08-31

**Authors:** Christine Cuskley, Rebecca Woods, Molly Flaherty

**Affiliations:** Language Evolution, Acquisition and Development Group, Newcastle University, Newcastle upon Tyne, UK; Department of Psychology, Davidson College, Davidson, NC, USA

**Keywords:** language evolution, language acquisition, writing, large language models

## Abstract

Researchers have recently argued that the capabilities of Large Language Models (LLMs) can provide new insights into longstanding debates about the role of learning and/or innateness in the development and evolution of human language. Here, we argue on two grounds that LLMs alone tell us very little about human language and cognition in terms of acquisition and evolution. First, any similarities between human language and the output of LLMs are purely functional. Borrowing the “four questions” framework from ethology, we argue that *what* LLMs do is superficially similar, but *how* they do it is not. In contrast to the rich multimodal data humans leverage in interactive language learning, LLMs rely on immersive exposure to vastly greater quantities of unimodal text data, with recent multimodal efforts built upon mappings between images and text. Second, turning to functional similarities between human language and LLM output, we show that human linguistic behavior is much broader. LLMs were designed to imitate the very specific behavior of human *writing*; while they do this impressively, the underlying mechanisms of these models limit their capacities for meaning and naturalistic interaction, and their potential for dealing with the diversity in human language. We conclude by emphasising that LLMs are not theories of language, but tools that may be used to study language, and that can only be effectively applied with specific hypotheses to motivate research.

## INTRODUCTION

Recent work has argued that large language models’ (LLMs) impressive ability to generate text (or its specific mistakes or limitations) can provide insights into how human linguistic cognition works. For example, Contreras Kallens et al. (2023) suggest that LLMs’ striking success shows that “grammatical language can be acquired without the need for a built-in grammar” (abstract). They argue that the performance of LLMs, which are designed to mimic human writing, is relevant evidence in longstanding debates about the roles of nature and nurture in human language (see Pleyer & Hartmann, 2019 for a detailed overview of these debates). They, and others (e.g., Piantadosi, 2023), make this argument in contrast to theories which propose that domain-specific, often “innate” capacities are essential to human language acquisition (thus requiring a built-in grammar). Simultaneously, proponents of this latter position focus on specific shortcomings in LLM performance, arguing that these provide support for domain-specific accounts (Chomsky et al., 2023), or at least leave these accounts with more explanatory power (Katzir, 2023,^1^; in response to Piantadosi, 2023).

In this paper, we take issue with the premise that the success of LLMs in generating (un)grammatical text is relevant evidence in this debate. Grounding our arguments in an ethological framework, we argue that the performance of LLMs only holds narrow relevance in better understanding the nature of human language. We argue this is particularly the case where the objective of cognitive scientific work is ultimately to develop a fuller understanding of the neurophysiological mechanisms, development, and evolution of language in humans.

We use a framework originally developed in ethology—the study of complex behavior in animals—to understand the relationship between human language and LLMs. This framework asks four key questions of behaviors: how does it develop, how does it work, how did it evolve, and what is it for? These four questions were specifically articulated by ethologist Niklas Tinbergen (1963), with significant influence from biology (Mayr, 1961) and longstanding ideas in philosophy (e.g., Aristotle’s four causes; Falcon, 2023). This four questions framework has been widely applied across biology (e.g., Strassmann, 2014) and psychology (e.g., Badcock et al., 2019), and is especially well-suited to understanding traits which arise as the result of complex interactions between biology and culture (Bateson & Laland, 2013). Language is the poster child for this kind of trait (Scott-Phillips et al., 2011; Spike, 2017): although debates about the relative role of biology (nature) and culture (nurture) in language abound, scholars generally agree that the devil is in the details. The question is not nature *or* nurture, but what the relative contributions of biology and culture are to human language, and how these interact in development (Pleyer & Hartmann, 2019). We work from these four questions to illustrate the limits, and possibilities, of the explanatory power of LLMs for understanding how human language and cognition work^2^.

First, we outline the broad details of this framework, using flight as an example of a complex animal behavior of which we have a relatively detailed understanding, and which also has an analogue in artificial flight. We draw a connection between artificial flight and artificial text generation of LLMs, and argue that the explanatory power of LLMs within this kind of framework is primarily limited to similarities in terms of ***functionality***: *what* they can do. Second, we explore limits to these functional similarities, outlining specific ways in which human language is a much broader and more complex phenomenon than the behavior exhibited by LLMs. We conclude with two specific precautions. First, we caution that functionally impressive technology can only reveal insights about human language when used with precision as a tool to reveal such insights, pointing to some ongoing work which does this effectively. Finally, we reiterate that excitement about a functionally impressive tool, while certainly warranted from a technological perspective, should not be mistaken for scientific revelation about human behavior.

## UNDERSTANDING COMPLEX TRAITS: THE FOUR QUESTIONS

As described above, the framework we adopt here asks four key questions about a complex behavior. While we aim to explain this framework in some detail here, we refer the reader to Bateson and Laland (2013) for a more detailed discussion. We can investigate the evolution of a trait in deep time (how did it evolve?), and its current utility or function in an organism (what is it for?). We can also look at how a trait works: its development over the lifespan of an organism (how does it develop?), and the physiological mechanisms that drive the behavior (how does it work?). This framework allows us to ground our questions in the behavioral trait we are seeking to explain (how does human language work), rather than hypotheticals related to that trait (could some computational system learn something like language given unconstrained computational power and input). This is not to say that hypothetical questions are not useful^3^, but to ground our discussion in specifically understanding human language. Below, we begin by illustrating the framework using the complex behavioral trait in birds of wing assisted incline running, for which we have a good understanding of the answers to each of these questions.

### A Well-Understood Trait: Wing Assisted Incline Running

**Figure 1.**
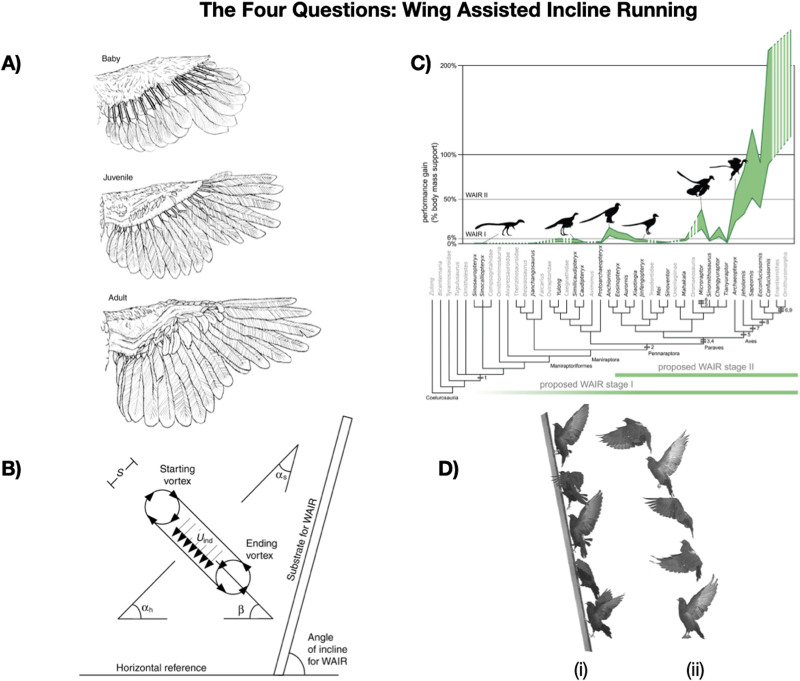
Representations of A) development (Tobalske & Dial, 2007), B) mechanisms (Tobalske & Dial, 2007), C) evolution (Dececchi et al., 2016), and D) function (Jackson et al., 2011). A, B and D adapted with permission from the Journal of Experimental Biology. C adapted under CC BY 4.0 License.

Below, Figure 1 illustrates the four questions for wing assisted incline running^4^, a form of vertical flight in which a bird uses its wings to help it scale a vertical surface using less energy than full aerial flight (Dial, 2003). Developmentally, flapping behavior develops relatively early in the lifespan, and the *development* of the individual’s feather morphology supports increasing ability to direct aerodynamic force onto a surface with flapping (Panel A; adapted from Tobalske & Dial, 2007). The *mechanism* behind the behavior involves running while gripping onto a vertical surface, using aerodynamic forces created by the wings directed towards the inclined surface (Panel B; adapted from Tobalske & Dial, 2007). In ultimate terms, the behavior *evolved* as a precursor to full aerial flight in dinosaurs, and acted in part as a selective pressure for the kind of aerodynamic wing morphology used for flight. This was an exaptation: selection modifying an existing trait into an adaptation with a new function (Gould & Vrba, 1982). In this case, feathers originally adapted for thermoregulation were exapted for full aerial flight (Panel C; Dececchi et al., 2016), potentially as part of the emergence of wing assisted incline running (see e.g., Benton et al., 2019). Finally, the behavior *functions* to allow birds to escape predation more rapidly by scaling vertical surfaces using significantly less energy (Panel D, i) than immediately undertaking aerial flight (Panel D, ii, Jackson et al., 2011).

Importantly, the four questions constitute four distinct research formulations for studying wing assisted incline running. No two questions are redundant: knowing how WAIR develops in young birds is distinct from understanding how the organism executes the behavior in their daily life. One could understand every step of the developmental process by which the trait manifests developmentally, and have little understanding or interest in the mechanism by which the behavior is executed. Understanding how the trait or behavior arose over evolutionary time is distinct from the question of what function the trait has for the individual in the present. A trait or behavior may arise in response to a specific set of evolutionary pressures but then be exapted for another modern day function. At the same time, while these questions may be distinct in terms of research motivations, that does not mean they are unrelated: insights into a behavior’s development may inform hypotheses about the mechanisms, for example. While information will necessarily flow between these questions, they can nonetheless be considered distinctly.

### The Four Questions for Human Language

**Figure 2.**
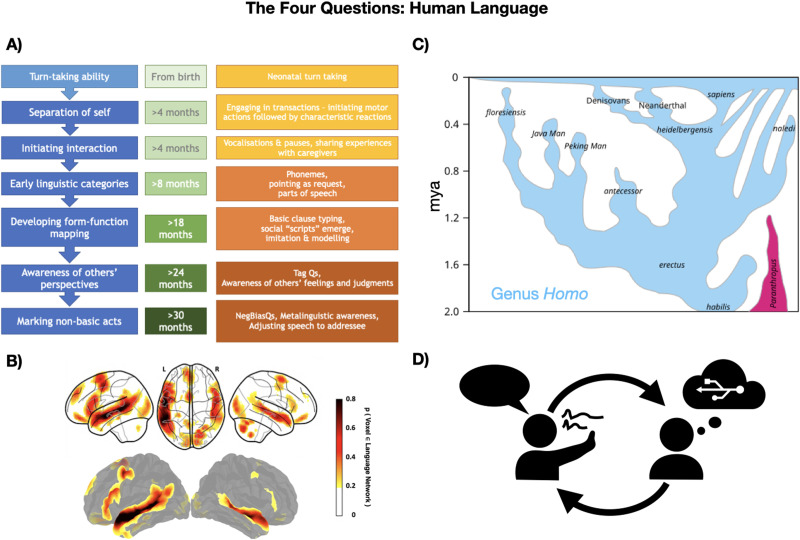
Representation of A) development (adapted from Woods, 2023), B) mechanisms (Lipkin et al., 2022; CC BY 4.0), C) evolution (Dbachman, Wikimedia Commons, CC BY SA. Hyperlink: https://commons.wikimedia.org/wiki/File:Homo_lineage_2017update.svg), and D) function (created by the authors) for human language.

While the workings of a trait like wing assisted incline running are fairly well-understood, the complexity and diversity of human language behavior, including the biological and cultural interactions involved, make this a much thornier trait to understand. Though considerable disagreement remains across the wide range of disciplines that investigate language, we do have some relevant information that can address each of the four key questions (Figure 2). Over the last half century, research in the development of language in the individual child has yielded rich insight into the process as well as uncovered extensive ground left to explore (Figure 2A). We know more than ever about the neurological mechanisms involved in language (e.g., Lipkin et al., 2022). The adaptive function of language is a matter of dispute; however, the main candidates—computation and thought (e.g., Hauser et al., 2014), versus communication and cooperation (e.g., Richerson & Boyd, 2010)—are by no means mutually exclusive. Finally, the evolutionary history of language is unfortunately not investigable to a large degree because unlike other aspects of cognition (see e.g., Scerri & Will, 2023), language does not leave a fossil record. Evidence points to some potentially language-like behavior in our last common ancestor with Neanderthals and Denisovans (Cuskley & Sommer, 2022), and also to a constellation of both homologous and analogous related capacities in species ranging from other apes to songbirds (Fitch, 2010). However, since language does not leave fossils the way feathers do, the question of how language evolved is likely to remain a matter of debate.

### The Four Questions for LLMs

**Figure 3.**
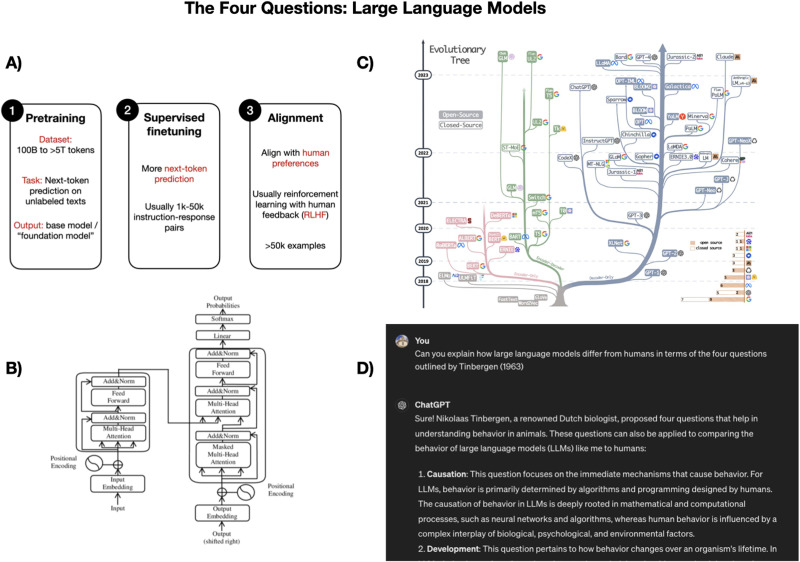
Representation of A) development (adapted with permssion from Rashka, 2023), B) mechanisms (Yuening Jia, Wikimedia commons, CC BY SA 3.0. Hyperlink: https://commons.wikimedia.org/wiki/File:The-Transformer-model-architecture.png), C) Evolution (adapted from Yang et al., 2023, CC BY 4.0), and D) function (Cuskley, custom GPT4 prompt).

Our understanding of the four questions in LLMs is considerably more complete. Given that they were designed by humans within the last few decades (Figure 3), we have considerable detail regarding how they work (although see Zhao et al., 2023), how they “develop”, and what technologies preceded them historically. Human language and LLMs do not share an evolutionary history: the cultural phylogeny of transformer models is a mere blip in the course of human evolution. These models were intentionally designed to accomplish specific tasks with their roots in the last 50–100 years of computer science, while human language naturally emerged in response to selective pressures at minimum, tens of thousands of years ago and possibly much earlier (Cuskley & Sommer, 2022). Human language and LLMs do have significant similarities in terms of their functional properties (Figure 2D, Figure 3D): both are used for communication and computation, and both can use text, though human language expands far beyond this function, unlike LLMs. In short, considering human language and LLMs within this framework establishes a null hypothesis that these things are, by and large, different from each other. As such, claims about their similarities require specific and targeted evidence. We will return to functional similarities—and their limitations—in The Limits of Functional Similarity section. First, we turn to establishing that development and mechanisms in human language and LLMs have fundamental differences.

### Development and Mechanisms

In terms of development and mechanisms, there is no overlap between human language and LLMs: LLMs develop using very different input and on very different timescales, and by fundamentally different mechanisms. We first address development and then move onto mechanism(s).

The conditions of development for human language learners versus LLMs differ drastically. Speech or sign signals received by human language learners contain rich information that is not straightforwardly encoded in the kind of text input that drives LLMs, including prosody, gesture, and world knowledge (e.g., Crystal, 1973; Speer & Ito, 2009). The developmental process of LLMs involves exposure to only a small slice of the human linguistic world: human (principally adult-to-adult) writing. In contrast with LLMs, human learners have a rich swathe of data available to them during language development. Researchers in the field of language development are currently engaged in the enterprise of identifying and quantifying this richness, but there is little dispute that this is qualitatively different from the text-only training received by LLMs.

The developmental process that child learners go through also divides into a developmental trajectory for comprehension and one for production. As production feeds comprehension (de Villiers & Pyers, 2002; Pierrehumbert, 2003; Vihman & Velleman, 2000) but comprehension (and proprioception) also, clearly, feed production (DePaolis, 2006), there are points in overall language development where production and comprehension do not necessarily track (e.g., scalar implicatures; Stiller et al., 2011). This is often because extralinguistic developmental processes, whether cognitive or physiological, also impact upon the development of both production and comprehension. As a result, the developmental trajectory is non-linear, and contingent upon multiple subprocesses and growing banks of knowledge and experience.

LLMs are explicitly designed to mimic text produced by human writers^5^, and as such, their developmental trajectory is significantly more linear, with fewer processes interacting to shape it, and less temporal variability in its underlying mechanisms. While the text generated by an LLM is not deterministic—part of what makes LLMs impressive is their ability to generate novel text, even when using identical prompts—their internal weights and mechanisms are generally static within a model version. On the other hand, child learners’ utterances are the product of a dynamic, constantly changing system: their utterances may vary moment to moment based on factors that do not feature in LLM algorithms, such as joint attention (Tomasello & Farrar, 1986; Tomasello & Todd, 1983) or extra linguistic factors, such as fatigue or general attentional capacities. These differences limit the *prima facie* explanatory power of LLMs for questions about how human individuals learn language and how language works in the human brain.

Consequently, LLMs cannot contribute to longstanding “nature/nurture” debates about human language, which generally revolve around innateness and domain specificity. Roughly, innateness^6^ can be defined as the extent to which aspects of language development are genetically or biologically predetermined, vs determined by learning and culture. Domain specificity is the extent to which the mechanisms underlying language learning and use are specific to language, as opposed to more general cognitive capacities. In other words, these are fundamentally debates about questions of development and mechanisms. Thus, LLMs designed to mimic functional aspects of human writing cannot immediately provide relevant evidence. The response of OpenAI’s GPT models (or LLaMa, or any other impressively performing LLM) to a prompt (e.g., used as evidence in Chomsky et al., 2023; Contreras Kallens et al., 2023; Piantadosi, 2023) is not relevant evidence in debates about development and mechanisms in human language work. We *know* that development and mechanisms in these two systems differ.

LLMs do have potential as a tool for discovery. On its own, technology designed primarily for the purpose of generating text holds limited relevance for questions about the neurophysiological mechanisms, development, and evolution of human language. But like many things developed for some other purpose, this technology can be usefully modified and applied to wider problems. Research using LLMs may help us to shape more useful research questions that can then be tested in humans, but are also essential in furthering our understanding of the functional capabilities of artificial intelligence. We return to the effective use of LLMs as a research tool in the Where Can LLMs be Useful? section. First, we further unpack our arguments for why LLMs are not a valuable source of insights when used more uncritically as evidence.

### What Can Artificially Designed Systems Reveal About Naturally Evolving Ones?

Studying the output of text-generation LLMs to learn about human language is a bit like studying a Cessna to learn about flight in birds. With carefully constructed, precise research questions, it may provide insights in functional, and perhaps broad mechanistic terms. We could, for example, tinker with a Cessna in specific ways to further our understanding of the effects of different air currents in Cessnas and birds. But this tinkering must be carefully and deliberately designed, and implemented to address specific theories and hypotheses that have demonstrated relevance to both phenomena (flight in Cessnas and in birds). We should not let the Cessna distract us from the study of bird flight itself, in all its aspects, if this is ultimately the behavior we wish to understand.

Understanding how flight works in birds is a very different goal from simply replicating its functionality by creating artificial flight; likewise, a full understanding of human language only overlaps partially with efforts to replicate certain functional aspects (in particular, text generation). In mistaking a new technological tool for a source of insight, we risk falling prey to a new iteration of the streetlight effect^7^: the urge to look for evidence only where we perceive the light to be most favourable, and not necessarily where the most enlightening answers are likely to be. For example, Krakauer et al. (2017), in writing about neuroscience, warn us of the ways in which technological innovation can lead to the abandonment of deep and difficult questions, instead shifting to questions which are more easily addressed with new technology. This risks “[sidelining] deep and thorny questions like ‘what would even count as an *explanation* in this context,’ ‘what is a *mechanism* for the behavior we are trying to understand,’ and ‘what does it mean to *understand* the brain?’” (p. 481). This is precisely the risk we face with LLMs and human language: technological innovation designed to mimic certain functional aspects of language should not determine the kinds of data we consider to be relevant evidence for understanding human language more broadly.

This does not mean that this technology (or any new technology) is not impressive, or that it can’t be useful for research purposes. Piantadosi (2023) and Contreras Kallens et al. (2023) argue that researchers have either claimed or implied that it was not possible for any entity to learn anything like language without some language-specific endowment. The achievement is impressive, and does indeed contradict this hypothetical argument. However, that some entity (a transformer model) can learn something like language (generate grammatical text) without some language-specific endowment does not have straightforward implications for how humans achieve this. Thus, in order for LLMs to be useful in understanding human language, they must be applied with surgical precision to specific questions in an intentional way. In Where Can LLMs be Useful? section, we return to emerging work that takes this approach for language: modifying open-source LLMs to probe specific questions about development and mechanisms in human language. Crucially, this entails an *a priori* testable framework (of the kind proposed in Krakauer et al., 2017) that is applicable to both human language and LLM behavior. Without such careful control, the point remains that language technology designed to mimic some functions of human language will not in itself shed meaningful light on questions about the evolution, mechanisms, or development of human language.

## THE LIMITS OF FUNCTIONAL SIMILARITY

To recap, LLMs and human language production are functionally similar in terms of grammatical text output, and increasingly impressive discourse coherence in text-chat style contexts. Having used the four questions framework to establish that similarities between human language and LLMs are functional, we now turn to the limits of those functional similarities.

### Meaning, Not Just Representations of Meaning

Many experts have noted functional limitations of LLMs in terms of meaning (sometimes framed in terms of understanding or knowledge; e.g., Bender & Koller, 2020; Mitchell, 2019; Mitchell & Krakauer, 2023). While some work seems to claim to show that LLMs have meaning, knowledge, or understanding at first glance, most of this work when examined in detail qualifies this in one way or another as being a *representation* (e.g., Li et al., 2021) or *approximation* (e.g., Piantadosi & Hill, 2022) of meaning. In other words, these representations or approximations of meaning give LLMs impressive functional performance, but this does not mean they have meaning in the same way humans do.

The distinction between meaning itself, and representations of meaning encoded in language, is a crucial element of over a century of work in semiotics. This work argues that meaning is derived from the *interpretation* of mappings between signs (forms) and signifiers (e.g., Peirce, 1977; Saussure, 1983; similar to Bender & Koller’s definition of meaning). Signifiers are things in the world that we are familiar with through embodied experience. LLMs do not have access to signifiers; they only have access to written linguistic forms, albeit a vast dataset of them. Without this perspective from semiotics, the ability to define a word using other words may seem sufficient to show knowledge and/or understanding of what it “means”. However, defining a form with other forms can be accomplished given sufficient access to forms and their relations to one another, irrespective of what they actually signify and evoke in the world. Because linguistic forms encode mappings between forms and signifiers for language users, the relationships between forms and the contexts in which they are used unsurprisingly hold some signature *representation* of meaning. In other words, language is itself a model of meaning in the world; as such, given vast amounts of language data, LLMs can build something like a model of the world, and very convincingly *mimic* meaning, even if it is impossible for them to actually *have* it. Indeed, many of the evolutionary precursors to LLMs, such as Word2Vec models (Mikolov et al., 2013) or Latent Semantic Analysis (Dumais, 2004) already leveraged the rich representations of meaning contained in grammatical text to create effective semantic NLP tools decades ago.

To further this point, consider a brief adaptation of a classic thought experiment derived from Turing’s Paper Machine (1951; and others, e.g., Searle, 1980), which we call the Spanish Dictionary. Spanish grapheme-phoneme mappings are rather consistent and transparent, unlike English, which is rife with inconsistencies (e.g., the different vowel sounds in *tomb*, *comb*, and *bomb* are all mapped to the same letter). In Spanish, however, the rules for how letters map to sounds are more predictable. Once you understand these basic mappings, and how to produce the associated Spanish phones, you can read almost anything aloud in a way that would be understandable to a fluent Spanish speaker. You could acquire these grapheme-sound mappings without understanding anything about what Spanish words mean, and in theory, run a service where you readily define any word for a Spanish speaker by looking up and reading entries out of a dictionary. You could do this without ever having any idea what your own utterances *mean* in the strict semiotic sense. The meaning is still there—and a fluent Spanish speaker will be able to access it readily—but as a person who only had a highly specific functional knowledge of Spanish *forms* (writing and pronunciation), you would not have access to it. In short, when we are impressed by the text generated by an LLM, it is because *we* are bringing the meaning to the table—the LLM can’t possibly have any idea what it is writing about in the sense that we expect a human to. Thus, an important limit to the functional similarity of LLM behavior and human linguistic behavior is that LLMs do not—and cannot—have meaning in a basic cognitive semiotic sense.

It is, of course, possible to disagree with the premise of this specific characterisation of meaning. Pavlick (2023) outlines arguments from conceptual role semantics, particularly from Harman (1982), who argues that “meaning is use, where the basic use of symbols is taken to be in calculation and not in communication” (p. 242). This echoes generative and usage-based disagreements about the functional nature of language particularly in relation to its evolution, where strong generativist arguments hold that language is an adaptation for computation (e.g., “language of thought”, Hauser et al., 2014, a term also used by Harman, 1982), while others argue it is an adaptation for communication (e.g., Richerson & Boyd, 2010)^8^.

Picking apart these issues at length is outwith the scope of this paper, but we note here that arguments from conceptual role semantics and similar theories, within a four questions framework, focus primarily on *function* (e.g., “meaning is use”): they aim to understand meaning primarily in relation to *what it does*, not how it develops in interaction with other cognitive mechanisms, or how it evolved. Yet, these issues are entailed in the *premise* of conceptual role semantics, where meanings are “determined by the concepts and thoughts they can be used to express”, and these are “determined by their functional role in a person’s psychology” (Harman, 1982, p. 242). Concepts, thoughts, and a person’s psychology come from their interaction with the world, regardless of whether they use meanings for calculation or communication. Thus, LLMs may have meaning if and only if we take a narrow functional definition of meaning, but not where we consider linguistic meaning in the full context of the four questions framework.

### Multimodality and Interaction

Recent research has made progress in building multimodal models that can deal not only with text, but also with data from other modalities, including images and video (Bubeck et al., 2023; Huang, Dong, et al., 2023), and all manner of other modalities that even humans generally cannot readily interpret (e.g., thermal data; Girdhar et al., 2023,^9^). Here it starts to potentially be the case, in cognitive semiotic terms, that models can map forms (text) to meanings (any other modality, such as images or thermal data). Such a model not only encodes relationships between text forms like uni-modal LLMs do, but also relationships between those forms and some other modality or modalities. However, the key issue is that even multimodal LLMs still lack any kind of organic symbol grounding (Harnad, 1990, 2024). While this is a limitation of LLMs and multimodal LLMs that use pre-trained text models, it is worth noting that there are other approaches which use a multimodal starting point. Vong et al. (2024) used in-situ audio and visual recordings from a 6–9 month old infant as training data for a neural network. They found that the network was able to engage in effective word learning, for example by identifying a specific object not in its training set (e.g., a ball it had never seen before as a ball; see also Orhan & Lake, 2024 regarding emergent visual representations).

In short, while a true system of meaning requires forms, meanings and mappings between those, these also must have some relationship to things in the world; the way in which (multimodal) LLMs work precludes this. We can return to the example of the Spanish dictionary to illustrate this by converting a text-only dictionary to be a look-up table for images. Knowing sound grapheme mapping for Spanish, one could look up a word, retrieve an image, and show that to whoever is querying the dictionary. Still, this could be done without our Spanish dictionary operator ever “knowing” or “understanding” what is in the image. They would only be able to “understand” this by virtue of their own experiences within the world.

While most language models are currently stuck on the point of having any real interaction with the world, this is not necessarily a key aim of most development of the technology. Resources are being devoted to developing AI to function in efficient ways that humans cannot, like rapidly generating realistic video footage based on a description (e.g., OpenAI’s Sora; Liu et al., 2024). Interaction with the world is nonetheless a major part of AGI efforts, and so embodied robots based on multimodal LLMs are on the horizon. For example, OpenAI and Figure One recently released a demo of a robot (reported in Drapkin, 2024) that was able to hand a tester an apple in response to a general request for something to eat, as well as to put away some dishes. While this is certainly an extension of functional similarity, humans simply do not work this way: the core developmental, mechanistic and evolutionary differences remain. No serious model of human cognition suggests that we develop first as vast closed networks of strings (or even signs or spoken words), only later opening our eyes, ears, mouths, noses, and reaching out with our hands to map our sensory input to linguistic forms.

It is precisely these mechanistic differences that place limitations on the technology’s ability to reach full functional overlap with human language. In the published demo, the speed of Figure One’s response to queries, while impressively fast for an assistive robot, is what marks it as functionally distinct from humans (note that many videos of this demonstration are intermittently sped up to mask these delays). Human conversation generally occurs with imperceptible delay between speakers at a rapid rate, with inter-turn delays of more than about 500 ms causing noticeable delay (Egger et al., 2010), and often includes overlap between speakers. Overlapping has multiple functions including collaboration (Schegloff, 2000) and repair (Kendrick, 2015). While LLM-based AI may close perceptible turn-taking gaps in conversation (i.e., reduce these to less than half a second), the mechanisms of LLMs will limit their ability to reach full conversational functionality. Unlike humans engaging in speech or sign, AI plans its entire utterance before it begins “speaking”. Phenomena such as pauses, hesitations and discourse markers (e.g., “um”) are often deliberately and stochastically built-in to speech models to make them seem more realistic (e.g., Betz et al., 2018), but they are not emergent. The model must convert speech requests into text, and then text into speech again before vocalising its response. It cannot, for example, clarify the aim of a request while the request is in progress, or modify its response as it is speaking based on the facial expression of the requester. Both of these are kinds of interactional repair, a phenomenon humans engage in frequently and fluidly in naturalistic conversation (Dingemanse & Enfield, 2024), and which plays a key role in effective communication. In short, the mechanisms of LLMs place limits upon its functional similarity with humans.

Functional similarity is also constrained by developmental differences. Even within the first two years of life, before they begin producing complex grammatically correct utterances, children display functional linguistic capacities that LLMs lack, and that interact in key ways with their development. For example, pragmatic knowledge and gesture play an important role in how children incrementally (but rapidly) build their grammars (Woods & Heim, 2023; Yang, 2022), with prosodic competence also feeding into development (though this is less well understood; Frota et al., 2014; Goodhue et al., 2023; Patel & Grigos, 2006; Pronina et al., 2022). These pragmatic, gestural and prosodic capacities are independently essential for achieving successful, functional linguistic interaction. Even in children as young as three, these functional capacities are already remarkably mature (Ambridge & Lieven, 2011; Lust, 2006; Rowland, 2013; Valian, 1986), while models lag behind (Weissweiler et al., 2023).

Even assuming models may eventually reach human-like functional competence in some pragmatic, gestural, or prosodic areas, the functional capacity for language in humans is not limited to the production of more or less well-formed strings for broadcast transmission. There are key functional elements of language that are not available to or emergent in LLMs, for example, turn taking (e.g., Casillas et al., 2016; Levinson, 2016; Stivers et al., 2009), co-speech gesture and multimodality (e.g., Goldin-Meadow & Brentari, 2017; Kita et al., 2007; Rasenberg et al., 2022), repair (e.g., Dingemanse & Enfield, 2024; Dingemanse et al., 2014, 2015; Hayashi et al., 2013), and common ground negotiation (e.g., Brennan & Clark, 1996; Brown-Schmidt & Duff, 2016; Clark & Wilkes-Gibbs, 1986) are all essential parts of natural language in interaction. This also blurs into other aspects of our broader socio-cognitive suite including ostensive inference, perspective taking and joint attention (e.g., Heintz & Scott-Phillips, 2023; Tomasello et al., 2005).

Even in infancy, as children just begin to distinguish between relevant sounds, language is not contained within the individual child. Young children co-construct their language along with caregivers and other interlocutors. Even “pre”-linguistic behavior, such as babbling, is multimodal and multi-directional. In the first year of life for example, infants use their babbles to regulate the complexity of their caregivers’ speech in ways that are especially helpful to their language learning stage (Elmlinger et al., 2019, 2023). Language functions as more than a series of broadcast transmissions; indeed, it was relatively early on in the history of widespread broadcast media that researchers found that linguistic input from radio or television broadcasts is not an effective form of input for child language learners (Sachs et al., 1981). Rather, children learn language from a complex, constant, interactive and multi-level symphony of coordination.

Notably, these differences interact closely with previously established differences in how children learn language (Development and Mechanisms section). This underscores the fact that how a system is acquired and works mechanistically will have consequences for what it can do functionally. Thus, many aspects of developmental and mechanistic differences lead to knock-on functional differences. What we do with language when we engage in complex turn taking and interaction not only contributes meaningfully to language development and acquisition in humans, but is something LLMs are not currently functionally or mechanistically capable of. Many key interactional aspects of linguistic form (and meaning) are emergent from interactions between language users, not confined properties of individual learners (Dingemanse et al., 2023). In other words, LLMs are designed as closed entities which fundamentally lack physicality, limiting their functionality in terms of embodied and interactional aspects of language.

What children—and thus, humans—*do* with language extends well beyond the text-generation limits of LLMs. Text generation in LLMs on the other hand, is often more impressive than in most humans—a serious concern with the technology is its ability to replace human writers effectively. In contrast, for humans, learning to decode texts and write effectively is an effortful process that requires explicit instruction. Unlike LLMs, which write linearly given the underlying mechanism of next token prediction, the process of long-form human writing in particular is fundamentally non-linear, and involves recursive revision (Lo Sardo et al., 2023). Moreover, developmentally, reading and writing emerge far later than spoken or signed language use and comprehension, and the timing of development is highly dependent on the writing system(s) used in the relevant culture—if such a system exists at all.

### Languages Beyond Text

The reliance LLMs have on using text input for training imposes several limitations in terms of their ability to be representative of human language writ large. First, this means that LLMs are unable to deal with human languages that are not or cannot be represented using text. Second, the reliance on text as part of a training pipeline for LLMs means it is not possible to adapt them to become representative of the broad diversity of human language (Atari et al., 2023). This may not be considered a problem for the technology: it can cover a vast number of users, even if it is only confined to a few common languages. However it *is* a problem for cognitive scientists, if we wish to use the technology as a source of evidence for answering questions about human language and cognition more broadly.

First, a reliance on text excludes an entire modality of natural human language: sign languages. Since the mid-20th century, the study of sign languages has profoundly deepened our understanding of human language by disentangling our complex communication system (and its cognitive underpinnings) from speech (Emmorey, 2001; Lillo-Martin & Henner, 2021; Stokoe, 1970). However, no sign language has a widely used written form. Deaf communities around the world fight for recognition and access to signed languages every day, in particular to prevent the dire (and common) consequences of childhood language deprivation (Hall et al., 2019). Encouraging an even narrower concept of language than the historical focus on spoken languages, LLMs equate language with text, fostering this misconception and further marginalising d/Deaf signers and signing communities.

Though some work has been done on sign languages and AI/LLMs, at this point in time this work is in its infancy. In a meta analysis of 101 papers on sign languages and AI methods, Desai et al. (2024), a team of Deaf and hard of hearing researchers, make several crucial points. They highlight that the field is dominated by hearing researchers who do not sign. While these researchers may have naïve good intentions to “solve” “problems” for Deaf and hard of hearing people, they often lack both the linguistic expertise to build appropriate tools, as well as cultural knowledge to understand the ways in which Deaf communities will be impacted by their work (see Hill, 2020 for related discussion of hearing researchers repeated misguided attempts to create “sign language gloves.”). NLP researchers have begun to recognize these concerns (see e.g., Yin et al., 2021), but at this point it is far from clear that these challenges will (or can) be adequately addressed.

Natural language without a written form is not unique to sign languages: language in face-to-face interaction emerged, at minimum, hundreds of thousands of years before writing systems (Lock & Gers, 2012)—indeed, literacy is a distinct complex behavioral trait that could have its own specific four questions. This is not a mere historical point: of the 7,168 living languages listed on Ethnologue, only a little over half (4,178) use a writing system (Eberhard et al., 2023). Most languages without a writing system are spoken languages. And in many cases where spoken languages do have a writing system, this was borrowed or adapted following colonisation rather than being designed (or emerging) for the language in question (e.g., the use of the Roman alphabet for Swahili). Even for languages and cultures with bespoke, established writing systems going back hundreds or thousands of years, widespread literacy is a phenomenon that emerged in most populations—including Europe and the US—only in the last century (Roser & Ortiz-Ospina, 2024). Natural languages, spoken or signed, emerge spontaneously in communities of users; in contrast, writing systems must be intentionally invented (or adapted), taught, and learned. In short, reading and writing (and thus, text) are themselves language technologies: indeed, much like linguistic forms create a model of meaning, writing is itself a (sometimes lossy) model of much more complex linguistic forms (Lock & Gers, 2012). Indeed, there are well-documented structural (including syntactic) differences even between transcribed naturalistic speech and written text (e.g., Biber, 1988). Although writing technology has been around much longer than LLMs, it is nonetheless only recently in widespread use in the longer context of human history.

Of the 4,178 spoken languages with a writing system, LLMs still only grapple with a fraction of this range, with the largest, BLOOM, covering 46 languages (Le Scao et al., 2022). Even these multilingual models exhibit uneven performance across languages: performance seems to scale with the size and quality of a training set, giving these models considerably superior performance in English relative to other languages (see Huang, Tang, et al., 2023, for a brief review). Over-estimating the relevance of LLMs to understanding human language and cognition risks further amplifying harmful existing biases towards English in cognitive science (Blasi et al., 2022). While it is the case that much of our current knowledge about human language is based on a small sample of languages, and often relies on text data as being representative of language more broadly, our argument is precisely that we should be more aware of the consequences of this. That some LLMs use other languages is only a patina of diversity; even where these show more or less equivalent performance to large models of English, we are still placing particular focus on only the written form of only (some of) the languages that happen to be written. This isn’t “merely” an issue of ethics and inclusion in scientific practice (a fundamental problem for AI that extends well beyond this; Bender et al., 2021; Birhane & van Dijk, 2020; Erscoi et al., 2023; Rillig et al., 2023), but risks leading us to only attempting to understand a confined subset of human linguistic cognition (Atari et al., 2023). Even if we confine our questions to very specific functional ones (e.g., whether LLMs are able to track long distance dependencies in utterances in the same way humans can), it is unclear how we can get answers that are broadly applicable to human language when this can only be tested for the kinds of languages that can be transcribed into vast quantities of text.

### The Implications of Functional Differences

The key functional differences outlined above—in meaning, multimodality, and overall modality—constrain the only area of concrete overlap between LLMs and human language to text production in some languages. Given a particular string of such grammatical text in one of these languages, it can be impossible to tell whether it was written by an LLM or a human. This has further implications for when (and whether) it is appropriate to adapt and apply LLMs to questions regarding the mechanisms, development and evolution of human language. If we wish to understand how human language works, how it develops, and how it evolved—but also, *everything* it can do functionally—using a tool only designed to generate text as a primary source of evidence builds in potentially undesirable assumptions about the object of study. By using LLMs as a key source of evidence about the nature of human language, we make the *de facto* assumption that the vast majority of language behaviors (speaking, signing, conversation, gestures, making shared meaning via linguistic systems, etc.) are not really language, or at least, are not meaningfully different from text.

One could run with this assumption, assisted by the general narrative of inevitability surrounding AI, and perhaps argue that the success of LLMs thus far (albeit on a small sample of written languages) indicates that they are likely to be able to deal with language in any form—it is only a matter of time. Maybe they learn in a different way, using different architecture, and maybe even learn a different thing entirely, for now—but they will get to other stuff later, increasing their explanatory power in terms of human language. However, this argument still rests on the flawed assumption that a biased, English-dominant sample of mainly spoken languages is representative of all human language (Blasi et al., 2022). This is the very assumption we explicitly challenge in arguing that LLMs are unsuitable for uncritically generating insights into how human language develops and works in the human mind. Although we acknowledge a focus on a relatively narrow sample of human languages has been the basis of progress in the field for much of the 20th century, we would argue (alongside others, e.g., Atari et al., 2023; Blasi et al., 2022) that now is the time to move more intentionally beyond this.

Moreover, LLMs have a fundamental mechanistic reliance on text that limits their ability to deal with spoken language input; in other words, the fact that LLMs can deal with speech in some languages is a facade. As discussed earlier, the ability of a model to interpret or generate speech relies on converting it to text. Automatic speech recognition models (e.g., Open AI’s Whisper) rely on training involving pre-mapped written transcripts and audio files^10^; unsurprisingly, transcript quality has been identified as a key factor in model performance (Radford et al., 2022). Like LLMs, speech-to-text transcription relies on using an existing writing system and training using large language-specific datasets. This means that the prospect of automatically generating usable text-based training data for low-resource languages via speech-to-text is unlikely even if they do have a written form, and virtually impossible if they do not. The scale of effort required to convert hundreds of thousands of hours of audio from an unwritten, low-resource language into something like the International Phonetic Alphabet (IPA) is infeasible, to say nothing of the issues inherent to collecting this much data from a low-resource language in the first place. The same limitations apply to a model’s ability to generate speech: artificial speech production, in everything from Siri to the latest multimodal release of ChatGPT, is based on text transcription.

These issues are compounded for sign languages, where no convention for consistently transcribing these languages (e.g., like the IPA for spoken languages) exists. Even if we had the hundreds of thousands of hours of video data necessary as a starting point, there is no format into which these could be converted that would allow us to train a language model. Overall, if we were able to direct intense efforts towards collecting vast amounts of data for low-resource (signed or spoken) languages, it’s not clear why these efforts would or should be directed toward building LLMs (instead of e.g., more deliberate and detailed efforts at language documentation, see Skirgård et al., 2023; as well as revitalisation, heritage and language justice efforts). Moreover, it is not clear that these language communities would *want* LLMs: there is a considerable history of technologists developing tools marginalized communities do not ask for, and without consulting them, resulting in “breakthrough technology” with little practical use (e.g., Hill, 2020). In short, to use technology as a comprehensive evidence for questions about human language, the mechanisms underlying LLMs (Figure 3A) would likely need to be radically rethought to deal more comprehensively with natural language forms.

These substantial concerns are unlikely to be relevant from an engineering perspective: the primary objective in designing LLMs was to generate realistic text and manipulate real text, in order to create technology likely to be adopted (and paid for) by the widest possible user base. From this perspective, the English-dominance of LLMs is a rational choice (e.g., at least half of the internet is written in English; W3Techs, 2024), and in this light, the fact that LLMs cannot deal with certain kinds of languages may not be perceived as a problem. Multilingual models that can perform competently in tens of languages are sufficient and even comprehensive from this perspective. In short, it might be argued that a narrow focus on some written languages isn’t a problem for LLMs (which is still not an uncontroversial claim, given uneven performance across languages). However, it should be considered an acute problem for cognitive scientists attempting to use LLMs uncritically as representative models of human language or cognition. One fundamental fact remains: no matter how impressive from an engineering perspective, these models were not designed to shed light on how language develops in humans, how it works in the brain, or how it evolved.

This doesn’t mean they cannot shed any light on these issues at all, but that this must be done carefully and precisely, and with appropriate caveats. It is possible to use LLMs as a tool to probe specific questions and test specific hypotheses relevant to the nature of human language. To conclude, we turn to examples of work pushing fruitfully in this direction.

## WHERE CAN LLMs BE USEFUL?

There is a massive, and constantly growing, body of work on better understanding the functional capacities and limitations of LLMs (e.g., Beguš, Dąbkowski, & Rhodes, 2023; Mahowald et al., 2024). However, in line with our earlier arguments, our premise is that this research (while inherently valuable) answers the questions outlined in Figure 3 (particularly Figure 3D). In other words, it aims to better understand LLMs themselves, not humans or human language. A key part of our argument is that for LLMs to be an effective tool for answering questions outlined in Figure 2, a more targeted approach is necessary.

### Structured Comparisons

Our detailed understanding of development in LLMs can allow us to probe specific questions about human development. As discussed in Understanding Complex Traits: The Four Questions section, the development aspect of LLMs is well understood in large part because these models were intentionally designed with relatively shallow roots in the last half century, rather than naturally evolving in deeper time. Particularly for open source models, such as LLaMa (Touvron et al., 2023), This means we have detailed information about the model’s architecture and training input. This gives us the opportunity to make structured comparisons between input and output in LLMs. The equivalent comparison for children is considerably more challenging: we lack a full picture of children’s input, and cannot ethically manipulate this to measure corresponding effects on their linguistic competence. Thus, LLMs give us an opportunity to test specific hypotheses about the relationship between input and output. Alongside carefully constructed comparisons to the behavior of child learners, this can yield new questions for investigation in children. Crucially, this is distinct from pointing to functional capacities in LLMs and drawing a straight line to conclusions about how children learn language.

Work in this vein has found that there may be systematic mappings between different grammatical elements and different layers of transformer models, and that verb conjugation may be encoded linearly in these models (Hao & Linzen, 2023), but also that surprisal in next word prediction does not do a good job of predicting difficulties in human syntactic processing (Huang et al., 2024). Kim and Smolensky (2021) find evidence that models represent abstract grammatical categories like nouns and verbs, and that it is able to create these categories from exemplars (Misra & Kim, 2023). These models can also demonstrate metalinguistic reasoning and competence (Beguš, Dąbkowski, & Rhodes, 2023) and produce recursive syntax when explicitly prompted to do so (Dąbkowski & Beguš, 2023). Nonetheless, for all of the aforementioned reasons, we are less convinced of the relevance of work comparing LLMs to human learning directly (e.g., Leong & Linzen, 2023; Yedetore et al., 2023), as the mechanisms remain profoundly different. Overall, while this work often acknowledges that developmental processes in these models are drastically different from human learners, it does not meaningfully address this limitation.

There is, however, ongoing work which aims to make more targeted comparisons between LLMs and child learners, focusing specifically on ecologically valid input. This work uses models trained on child directed speech, increasing ecological validity for questions related to child language learning. BabyBERTa (Huebner et al., 2021), for example, is trained exclusively on transcribed child-directed speech. This more naturalistic input is a substantial step up in terms of building models that may shed light on questions regarding the nature of human language. Crucially, however, the work focuses on comparing the performance of BabyBERTa to larger models trained on more traditional text from the RoBERTa family (see Figure 3C, top right, see also Yang et al., 2023; Gao & Gao 2023)—interestingly, BabyBERTa shows comparable performance to much larger versions of RoBERTa. However, the model is not compared directly to child performance. This has interesting implications for the richness of child-directed speech in particular, but this work is primarily informative about how human-like data can improve *models*, not about what children do and how they do it.

The BabyLM challenge (Warstadt et al., 2023a) expands even further upon this idea: teams of researchers competed to train language models on developmentally plausible datasets (in terms both using child directed speech as input, and constraining the size of the training set). Three tracks of the challenge each have different guidelines for the size of this English-only dataset, as well as the type of data on which the model would be trained. In the strict-small track, training corpora consisted of 10 million words (an upper limit of as much input as children might get in their first two years), in the strict track they consisted of 100 million words (potentially equivalent to how much input children may get in their first 10–12 years), and in the loose track the training corpora consisted of 100 million words plus an unlimited amount of non-linguistic data (including music, visual input, and audio language samples). The challenge received 31 entries, each choosing a track, and describing their models, training, and results.

Some of these models (e.g., the winning model in the BabyLM challenge, Samuel, 2023) were able to push mastery of complex syntactic phenomena past the performance of earlier models like the original BabyBERTa (Huebner et al., 2021). Other models in the challenge took on intriguing aspects of the problem not already addressed by the challenge. In particular, Steuer et al. (2023) confronted a crucial challenge for LLM work attempting to teach us about human language learning: processing effort and efficiency. Though the human language learner clearly faces limitations in terms of amount of potential processing effort and quantity of time available for learning, Steuer and colleagues point out that the BabyLM challenge did not include any criteria along these lines. They write that if work with LLMs is to inform understanding of human language learning, “it would be worthwhile to move in the direction of a unified approach that accounts for both forms of linguistic competence and empirical evidence of processing effort.” (p. 8). We wholeheartedly agree with this suggestion.

As Warstadt and colleagues point out, even the most impressive submissions to BabyLM have thus far failed to produce and understand basic interrogative clauses (and exchanges containing them; Warstadt et al., 2023b), while children can do this by age 2. These models have also thus far not demonstrated mastery of the kinds of open-ended text generation tasks that have so impressed many cognitive scientists (Contreras Kallens et al., 2023; Frank, 2023; Piantadosi, 2023). Precisely by examining these limitations, we may develop new research questions that delve into why exactly child-directed speech is a boon for child language learning, but not necessarily for LLMs. It is worth noting that what is revelatory about this work for human language is less where LLMs succeed, but where they fail.

Nonetheless, the fact remains that these comparisons are limited: the scale of training data LLMs require in development dwarfs the number of tokens human learners encounter^11^. Crucially, even the way the size of the training set is conceptualized is fundamentally different from the realities of child language input. For example, the BabyLM Challenge limited only the size of the training set, not the number of times that set was presented to the model (the number of training epochs). The most developmentally plausible data sets in quantitative terms, such as those requiring a similar number of input tokens to a child’s first two years of language learning (approximately 10 million words), still use multiple (and often hundreds) of training epochs in order to achieve reported model performance; this means the model passes over the training set many times. Children encounter about 10 million words in total by the age of two; not the same 10 million words tens or hundreds of times. This means the model is trained on the amount of language data a child might receive in the first two years many times over. Although the data is qualitatively based on the kinds of words a child receives in their first two years, if trained for 100 epochs, it is quantitatively equivalent to the input of hundreds of children presented sequentially. While epoch training is a standard approach for computational models, this is a fundamental developmental difference between models and child learners. Training sets based on child directed speech are certainly *more* ecologically valid than unconstrained training sets used for many LLMs, but quantitative and mechanistic differences in training make precise comparisons difficult.

In abstract information theoretic terms, the total input human learners receive across their much richer multimodal experience extends beyond a mere count of the total number of tokens they receive. But crucially, we don’t understand exactly how, or how much. Unless and until we have a better understanding of the total value of this input, it’s not yet possible to make complete comparisons between the input humans receive and the input received by LLMs. Nonetheless, this is an interesting avenue to pursue. Children’s overall input could, in theory, be closer in scale to that of LLMs; though we are currently unable to even attempt to quantify this without a fuller understanding of all the dimensions of input children use in language learning (see Frank, 2023 for additional discussion of some ways human language input is richer than that of LLMs).

Though working with older models, Schrimpf et al. (2021) provide a roadmap of the type we’d like to see in terms of how to learn more about human language using large language modelling. Echoing Box’s (1976) aphorism in statistics, the authors write, “Given the complexities of the brain’s structure and the functions it performs, any one of these models is surely oversimplified and ultimately wrong—at best, an approximation of some aspects of what the brain does. However, some models are less wrong than others, and consistent trends in performance across models can reveal not just which model best fits the brain but also which properties of a model underlie its fit to the brain, thus yielding critical insights that transcend what any single model can tell us” (p. 2). Importantly, they emphasise that this work must involve comparing the results from many models to see which most closely resemble data from human linguistic behavior. While Schrimpf and colleagues discuss language processing (i.e., mechanisms) in their paper, this approach could also be appropriate for questions of language learning (i.e., development).

### Probing Specific Functional Capacities

Computational models do have the potential to teach us about human language learning, however, it’s not clear that LLMs are the best source of insights. While every problem may look like a nail to the hammer of LLMs, the computational resources required by these models, and the corresponding carbon footprint (Rillig et al., 2023) make these a questionable first port of call for insights about language. Here, we revisit successful efforts to intentionally model human language which take a more modest approach.

Half a century of work in child language learning (e.g., Brown, 1973; Bybee & Slobin, 1982; Figueroa & Gerken, 2019; Marcus et al., 1992; Slobin, 1971) provides us a rich picture of how English learning children acquire the English past tense. In learning the past tense irregular English verbs, children first produce correct irregular forms (i.e., “broke” for the past tense of “to break”), then begin to over-regularize the regular -ed English verb ending (i.e., producing “breaked” instead of “broke”), before eventually using the verb in an adult-like way^12^ (again producing “broke”). This is generally known as U-shaped learning: performance dips in development before recovering again as children learn rules *and* their exceptions.

LLMs that seek to model child learning focus on large training sets, billions or trillions of parameters, and the general task of next word prediction. Modelling the changing grammatical competence of the child learner over development, however, requires more than next token prediction, and has the potential to tell us more about how human language learning may work. Harking back to the pathbreaking modelling work of Rumelhart and McClelland (1987), much more efficient models have successfully probed important questions about the relationship between rules and exceptions in language, using the English past-tense as a test case (Plunkett & Juola, 1999; Plunkett & Marchman, 1993; Taatgen & Anderson, 2002). Crucially, these models are built with the aim of reproducing typical child language learning errors (e.g., U-shaped learning) as a natural consequence of their architecture and training. Aiming to replicate the kinds of errors children make, instead of aiming for error free output, makes these compelling models of child learners. Other more recent approaches uses smaller neural network models on multimodal data, aiming to use data collected from children’s linguistic experience to probe what models can (and cannot) learn from these datasets (e.g., Vong et al., 2024; Wang et al., 2023).

Interestingly, when we do give LLMs these kinds of specific functional tasks, they don’t necessarily perform well. For example, given a Wug-task that tests the generalisation of morphological rules to novel stems. Weissweiler et al. (2023) tested ChatGPT against several more modest computational models designed to look specifically at morphological learning. Across English, Tamil, Turkish, and German, more modest models generally showed much more impressive performance. We do not aim to argue that LLMs have no potential as cognitive models; rather, we suggest that this potential can only be realized where LLMs are specifically designed or adapted to model particular cognitive phenomena. In short, if we wish to model next word prediction, LLMs are perhaps a reasonable solution; but where we wish to model how learners generalise morphological rules, a more targeted approach is warranted. Where we have specific questions about particular linguistic phenomena—and how they work in humans—smaller, more targeted models are a more efficient solution with greater explanatory power.

## CONCLUSIONS

Human language is a complex behavioral trait. We can investigate how it develops in the individual’s lifespan, what its underlying mechanisms are, how it evolved, and what its current function is. Questions related to language function—at least if these are limited to text—may be fruitfully investigated using LLMs, but those questions must be carefully constructed. However, LLMs are entities that are ontologically distinct from humans, and cannot, by their mere existence, inform us with regard to three of these questions: how language evolved, how it develops in the (human) individual, and how it works in the human brain.

Any potential LLMs might have to push our understanding of human language and cognition forward substantially is unlikely to be realized without a more comprehensive understanding of the data humans use in language learning, radical transparency surrounding the training sets and architecture of LLMs, and serious consideration of language diversity. We are not recommending the end of language research using LLMs. Rather, we argue that LLMs are not the answer to a question; they are a tool that we can use to potentially understand some specific aspects of human language. Crucially, this can only be effective if we apply this tool critically, acutely aware of its limitations, and with specific theoretical motivations. Indeed, regardless of tools or methods we apply in our research, we should aim to have well-articulated theories before we subject them to empirical tests (van Rooij & Baggio, 2021).

We argue this is particularly true of key questions at the heart of the cognitive science of language in the last half century, regarding interactions between the role of culture and biology in language, and the ways in which these dovetail with the domain specificity or generality of language. Incidentally, the authors have mixed views on this issue: Cuskley and Flaherty work in more usage-based traditions, while Woods’ work frames investigations into language acquisition in the generativist tradition. In all, while we occupy different parts of the spectrum of this debate, none of the authors takes an especially extreme stance; we generally emphasize interactions between input and biology, and if we disagree on domain specificity or generality, this is a matter of *degree*. As such, our arguments here are not in service of using LLMs to advance this debate in one direction or the other. Rather, our key argument is that LLMs alone cannot meaningfully advance this debate.

In summary, LLMs are not designed to provide particularly strong or weak support for or against any particular theory of human linguistic cognition (nor do they incidentally provide insights in this domain); LLMs are not theories, they are tools. They have narrow functional similarity to written language: they can generate syntactically well-formed text in some languages. However, their reliance on (and confined functional competence in) text, the necessity of massive training sets, and the ways in which they are passively trained, mean they are fundamentally different from humans in non-trivial ways. Their performance (or lack thereof) on text generation tasks cannot contribute meaningfully to debates about the extent to which human language learning is domain general or domain specific, or whether language involves neurological structures that are “innate” or merely develop robustly given adequate input. LLMs are fundamentally divorced from the vast array of behaviors, and broad base of cognition, that are tightly tied to human language.

## ACKNOWLEDGMENTS

We thank reviewers for detailed comments, critique and engagement which greatly improved the work.

## FUNDING INFORMATION

This work was supported in part by a British Academy Newton Alumni Fellowship (NA23\100009) awarded to Molly Flaherty.

## AUTHOR CONTRIBUTIONS

C.C.: Conceptualization; Project administration; Visualization; Writing – original draft; Writing – review & editing. R.W.: Conceptualization; Writing – original draft; Writing – review & editing. M.F.: Conceptualization; Funding acquisition; Writing – original draft; Writing – review & editing.

## DATA AVAILABILITY STATEMENT

This work does not draw on analysis of any specific dataset; as such no data are made available.

## Notes

^1^ Katzir (2023) makes some arguments similar to ours, e.g., drawing comparisons between LLMs and artificial flight (which appeared in our own drafts in late 2022), however, he nonetheless proceeds (in response to Piantadosi, 2023) to use the particular behaviors of ChatGPT as “empirical tests of adequacy, showing that LLMs fail on all of them.” In other words, the bulk of the work focuses on using the model’s behavior to test a particular theory about human language.^2^ Note that where we refer generally to “human language and cognition”, or the “nature of human language”, we take this to encompass the sum of (and interactions between) all four of these areas, identified in more detail in Understanding Complex Traits: The Four Questions section.^3^ This kind of question may be more of a focus where the primary aim is to functionally replicate certain aspects of language or human behavior, as is generally the aim of LLMs and AI more broadly. However, see Lake et al. (2017) for a detailed argument as to why AI engineering should consider developmental and mechanistic aspects of human behavior.^4^ Note that we do not aim to give a comprehensive overview or explanation of flight in birds here, and indeed, there likely remain gaps in our understanding of this trait, which are being filled by ongoing research. However, relative to many complex human behavioral traits (e.g., language), flight is fairly well understood.^5^ This is not always the case; some LLMs are trained on datasets generated by other models, rather than by humans. However, it is worth noting that this sometimes causes a marked decay in model performance (Shumailov et al., 2023).^6^ We use “innateness” here because this concept is widely used to frame this debate; however, see Mameli and Bateson (2011) for a discussion of why the concept of innateness is often ill-defined and limited in its usefulness.^7^ The streetlight effect is a well established issue in the sciences where researchers are biassed towards evidence that is more accessible, even if it is not necessarily relevant. This is often illustrated using the anecdote of someone searching in a parking lot for their lost keys in the well-lit glow of the streetlight, rather than the darkened side of the parking lot where they actually dropped them.^8^ As noted in The Four Questions for Human Language section and elsewhere, the authors fail to see how these two arguments are necessarily mutually exclusive, despite the fact that they are often framed as such. It is not unusual for complex traits to have multiple functions (e.g., feathers are essential for flight, but are retained in flightless species for thermoregulation).^9^ Note, however, that even these impressive multimodal models—which may have emergent multimodal mappings (i.e., develop mappings on which they were not explicitly trained)—use pre-trained image and language models e.g., DALL-E and CLIP in the case of Girdhar et al. (2023). This underscores that, regardless of increasing functional similarity, they remain developmentally and mechanistically distinct.^10^ This is not to say that no computational models are trained on raw speech data (e.g., see Beguš, Lu, & Wang, 2023), but that ASR generally in daily use, and as implemented with most LLMs, involves transcribed text as a key part of its training.^11^ It is also the case that expanding the training set size of LLMs can result in their output resembling human language performance *less* well (see McKenzie et al., 2023; Oh & Schuler, 2023), which is qualitatively unlike any findings in humans.^12^ It is important to recognize that the adult-like form may not be agreed upon by 100% of adult speakers, nor used entirely consistently by an individual adult. For some verbs (i.e., *shine* and *shined* or *shone, sneak* and *sneaked* or *snuck*), both forms may be acceptable even within a single individual (Cuskley et al., 2014, 2015).
